# Tumoral pSMAD2 as a prognostic biomarker in early-stage breast cancer: insights from the randomized SweBCG91RT trial

**DOI:** 10.1007/s10549-025-07744-0

**Published:** 2025-06-09

**Authors:** Axel Stenmark Tullberg, Viktoria Thurfjell, Anikó Kovács, Patrick Micke, Aristidis Moustakas, Fredrika Killander, Emma Niméus, Erik Holmberg, Per Karlsson, Carina Strell

**Affiliations:** 1 Department of Oncology, Institute of Clinical Sciences, University of Gothenburg, Sahlgrenska University Hospital, 41345 Gothenburg, Sweden; 2 Department of Immunology, Genetics and Pathology, Uppsala University, Dag Hammarskjölds V 20, 751 85 Uppsala, Sweden; 3 Department of Clinical Pathology, Sahlgrenska University Hospital, Gothenburg, Sweden; 4 Institute of Biomedicine, Sahlgrenska Academy, University of Gothenburg, Gothenburg, Sweden; 5 Department of Medical Biochemistry and Microbiology, Science for Life Laboratory, Biomedical Center, Uppsala University, Uppsala, Sweden; 6 Division of Surgery and Oncology, Department of Clinical Sciences, Lund University, Lund, Sweden; 7 Department of Surgery, Skåne University Hospital, Lund, Sweden; 8 Centre for Cancer Biomarkers CCBIO, Department of Clinical Medicine, University of Bergen, Bergen, Norway; 9 Division of Oncology, Department of Clinical Sciences, Lund, Lund University, Skåne University Hospital, Lund, Sweden; 10 Department of Medicine, Stanford University, Stanford, California, USA

**Keywords:** TGF-β signaling, SMAD2, Breast cancer, Prognosis, Radiotherapy, Immune cells

## Abstract

**Background/Aim:**

The TGF-β pathway can influence breast cancer progression and therapy efficacy, exhibiting both pro- and anti-tumoral effects. This study examined the impact of active TGF-β signaling on recurrence and radiotherapy (RT) benefit in early-stage breast cancer, using nuclear phosphorylated Smad2 (pSMAD2) as a marker for pathway activation.

**Methods:**

Tissue-microarrays from 1178 stage I-IIA breast cancer patients in the SweBCG91RT trial (randomized to breast-conserving surgery with or without RT) were analyzed. pSMAD2 immunohistochemistry was scored as the mean percentage of tumor cells with nuclear staining. Recurrence risk and RT benefit were evaluated.

**Results:**

pSMAD2 scores were heavily skewed, with 45% of tumors demonstrating high staining (≥ 80% tumor cells), 38% medium (21–79%), and 17% low (≤ 20%). Low pSMAD2 tumors were associated with higher grade and larger size but not with subtype. Medium pSMAD2 tumors had a significantly increased ipsilateral breast tumor recurrence risk than high pSMAD2 tumors (HR_adjusted_ = 1.82, *p* = 0.002), while no differences were observed for low pSMAD2 tumors. A similar result was obtained with all recurrences as endpoint. RT benefit was consistent across all pSMAD2 groups. In Luminal tumors, higher tumoral pSMAD2 levels were inversely correlated with tumor-infiltrating lymphocytes (TILs).

**Conclusion:**

Medium pSMAD2 levels were linked to an increased recurrence risk compared to high levels, suggesting a tumor-suppressive role of TGF-β in early breast tumorigenesis. However, no significant differences were noted for low pSMAD2 levels. In Luminal tumors, TGF-β signaling was negatively associated with TILs. These findings indicate that therapeutic targeting of TGF-β warrants careful consideration of tumor stage and subtype.

**Supplementary Information:**

The online version contains supplementary material available at 10.1007/s10549-025-07744-0.

## Background

Dysregulation of TGF-β signaling has been implicated in nearly all cancer types [1]. In breast cancer, several preclinical experimental studies have established an association between TGF-β signaling and crucial aspects of tumor progression, metastasis, and immune evasion [2–5].

This complex pathway is governed by the transforming growth factor (TGF)-β superfamily of 33 human cytokine genes, which exert pleiotropic effects during tissue development but also during adult tissue homeostasis and regeneration (reviewed in detail [1, 6, 7]). Within this superfamily, three TGF-β ligand genes have been identified: TGF-β1, −2, and −3. In the canonical signaling pathway, TGF-β ligands bind to hetero-tetrameric serine/threonine kinase receptors (TβRI and TβRII), leading to intracellular phosphorylation and activation of R-SMADs 2 and 3, which subsequently associate with co-SMAD4. The SMAD complex accumulates in the nucleus where it orchestrates the expression of a variety of genes, involved in the cell cycle, apoptosis, cell differentiation, fibrosis, and immune response regulation [8, 9].

TGF-β signaling exerts its different effects on diverse cellular functions in a context-dependent manner upon cell and tissue type [9, 10]. This highly contextual function gives rise to a complicated role for TGF-β ligands in cancer development, wherein they exhibit both tumorigenic and tumor-suppressive effects [11–13]. In normal tissue and premalignant lesions, TGF-β ligands demonstrate antiproliferative effects, while as cancers progress, TGF-β signaling takes on a more tumor-promoting role including the induction of EMT or tumor cell migration and invasion [1]. This switch is likely caused by the acquisition of mutations, copy number alterations or losses either in genes of the TGF-β pathway itself or its interconnected regulatory pathways [4, 14–16]. However, this switch is not yet precisely defined in terms of its temporal onset in patients. Various preclinical studies further revealed significant effects of TGF-β signaling on the tumor microenvironment, such as immunosuppression or fibroblast activation [17–21].

Consequently, TGF-β pathway antagonists are rapidly emerging as highly promising and effective anticancer agents, e.g., the small molecule galunisertib (inhibiting TβRI) or the monoclonal antibodies PF-03446962 (blocking TβRI) and fresolimumab (neutralizing TGF-β1,2,3) [22]. However, while the inhibition of TGFβ signaling has demonstrated promising outcomes in mouse models and in vitro studies, including breast cancer models, effectively preventing EMT induction and immune evasion [23–30], clinical trials have regrettably failed to convincingly replicate these findings across different tumor types. These disappointing results are likely due to the ubiquitous involvement of TGF-β-signaling in tissue homeostasis and the biological complexity of cancers encompassing diverse genetic properties, different disease stages, and a heterogenous tumor microenvironment [22, 31, 32]. This leads to challenges in identifying patients who can benefit from TGF-β inhibitors as the treatment may have unwanted tumor-promoting effects if administered in inappropriate settings [33]. Furthermore, these clinical setbacks underscore the need for additional clinical research regarding this important pathway. Overcoming the challenge of selectively blocking only tumor-promoting activities while preserving the tumor-suppressive effects of TGF-β demands a better understanding of its role in cancer biology in the clinical setting [32]. The clinical development and integration of TGF-β antagonists require novel biomarkers to better stratify patients and improve response rates [22].

In the context of breast cancer, analyses based on patient samples regarding the prognostic implications of TGF-β signaling have yielded disparate findings. Two independent studies observed that elevated tissue TGF-β levels were correlated with shorter disease-free survival and lymph node metastasis [34, 35], while conversely, two other studies reported an association between higher tissue TGF-β levels and favorable tumor features, including reduced metastasis [36, 37]. Notably, both of the latter studies highlighted that the loss of TβRII was linked to an unfavorable prognosis, albeit in a histological subtype-specific manner. A limited number of studies investigated the prognostic relevance of phosphorylated SMAD2 (pSMAD2) as an indicator of active TGF-β signaling in diagnostic breast cancer tissues, with both suggesting an adverse prognosis and an increased risk of disease recurrence [36, 38].

Investigations on ductal carcinoma in situ (DCIS, stage 0) have indicated that a shift toward a less active tumor immune environment occurs already at this early tumor stage and in a TGF-β-dependent manner, leading to a restricted infiltration of immune effector cells, accompanied by an increase in the number of regulatory T cells [39]. Additionally, in DCIS, a decrease in TGF-β and hedgehog signaling activity has been linked to the loss of myoepithelial cells, thereby contributing to tumor progression [4].

Collectively, these studies provide evidence supporting that the effects of TGF-β are likely dependent on the tumor stage and histological subtype as well as treatment modality.

In order to explore TGF-β signaling as a potential biomarker or therapeutical target it is therefore important to perform analyses in well-defined patient populations. The aim of this study was to investigate the impact of active TGF-β signaling on recurrence risk in early-stage breast cancer, including ipsilateral breast tumor recurrence (IBTR) and all recurrence types, with a particular focus on the benefit of radiotherapy (RT), using nuclear pSMAD2 as a biomarker.

## Materials and methods

### Study population

Patients from the randomized SweBCG91RT trial were included as described previously [40]. In summary, 1178 patients younger than 76 years with lymph node-negative stage I or IIA breast cancer were randomly assigned between 1991 and 1997 to breast-conserving surgery with or without whole-breast RT and followed until 2013 for a median time of 15.2 years. RT was given as tangential opposing beams with 4–6 MeV photons to a total dose of 48–54 Gy. The allocation ratio between RT and no RT was 1:1. A tissue microarray (TMA) was constructed from formalin-fixed paraffin-embedded (FFPE) blocks of treatment naïve tissues taking two 1 mm cores from the central part of each tumor, where the percent of tumor cells ranged between 50 and 95% (median 80%), as described previously [41]. Tumor subtyping was performed according to the St Gallen International Breast Cancer Conference (2013) Expert Panel on TMA slides as described previously [41]. Invasive carcinoma had histologically been confirmed by a board-certified pathologist. In total, 7% of patients received endocrine treatment, 1% received chemotherapy, and 0.4% received both endocrine treatment and chemotherapy. The study was conducted in accordance with the declaration of Helsinki. Informed oral consent was obtained from all patients, and the original trial and this follow-up study were approved by the Regional Ethical Review Board (approval numbers 2010/127 and 2015/548).

### Immunohistochemistry for pSMAD2

Freshly cut 4 um sections of the TMA blocks were used for immunohistochemical staining (IHC). The rabbit monoclonal antibody targeting phosphoSMAD2-Ser465/467 (Cell Signaling, #3108, clone 138D4) was employed at a dilution of 1:500 in BOND Primary Antibody Diluent (Leica Biosystems, #AR9352). The staining was executed on the Leica BOND RXm autostainer utilizing the Polymer Refine Detection kit with DAB (Leica Biosystems, #DS9800) including the default dewaxing procedure, antigen retrieval for 30 min at 100 °C with Epitope Retrieval Solution 2 (pH9, #AR9640), blocking of endogenous peroxidases, antibody incubation for 20 min at room temperature, staining development with DAB for 10 min and hematoxylin counterstain for 8 min.

For staining controls, FFPE embedded pellets of A549 cells were prepared, representing control/untreated, + TGF-β or + TGF-β + Ly2157299 (TβR1 inhibitor) (Supplementary Fig. 1A). The embedding followed the clinical procedures at the pathology unit at Uppsala University Hospital.

### Pathological annotation of pSMAD2 staining

Stained slides were scanned at 20× using the NanoZoomer S60 (Hamamatsu) slide scanner. Images were viewed and annotated in PathXL by a board-certified pathologist (VT) blinded to any clinical data. For all cores the tumor cell as well as stroma cell fraction positive for nuclear pSMAD2 was annotated using fixed incremental steps as follows: 0%, 1%, 5%, 10%, 20%, 30%, 40%, 50%, 60%, 70%, 80%, 90%, 95%, or 100%. Cores with < 50 tumor cells were not annotated. Only nuclear staining with presence of a brown tone involving 50% or more of the nucleus and distinct discrimination from the surrounding cytoplasm, was regarded as positive (Supplementary Fig. 1B–C). Finally, the pSMAD2 scores of the two cores per patient were averaged for the tumor and stroma compartment, respectively.

### Tumor-infiltrating lymphocyte scores and immune marker scores

Tumor-infiltrating lymphocytes (TILs) had been evaluated for the SweBCG91RT trial cohort on large-format hematoxylin–eosin-stained full sections, following the guidelines of the International Immuno-Oncology Biomarker Working Group for TIL assessment in breast carcinoma, as describes and published previously [42]. Evaluation of CD8^+^ T cells and FOXP3^+^ Tregs had been performed and reported previously, via single IHC on the TMA and evaluated as the proportion of TILs occupied by the respective cell type [43]. Additionally, the immune checkpoint markers PD-1 and PD-L1 had been stained by single IHC on TMAs and scored by two board-certified pathologists [44].

### Statistical analysis

The correlation between tumoral and stromal pSMAD2 status was evaluated using Spearman’s Rank test. Associations between clinicopathologic parameters including immune marker profiles and tumoral pSMAD2 status were analyzed using Anova test for continuous variables, and Pearson’s chi-squared test for categorical variables. Time to ipsilateral breast tumor recurrence (IBTR) or any type of recurrence (all recurrence) as first event within 10 years were analyzed as predefined end-points considering regional recurrence, distant recurrence, and death as competing risks. Cumulative incidences were calculated according to the method by Fine and Gray [45]. Hazard ratios (HRs) were calculated with uni- and multivariable cause-specific Cox proportional hazards regression. Only variables that were significant in univariable analysis were included in the multivariable model.

In order to assess a potential association between tumoral pSMAD2 status and radiotherapy benefit, an interaction term between pSMAD2 and radiotherapy was included in the Cox regression models. The proportional hazards assumption was tested with Schoenfeldt’s test. *p* values < 0.05 were considered significant. STATA 15.1 was used for analysis (StataCorp. 2017. Stata: Release 15. Statistical Software. College Station, TX: StataCorp LLC).

## Results

### pSMAD2 staining and evaluation

**Fig. 1:**
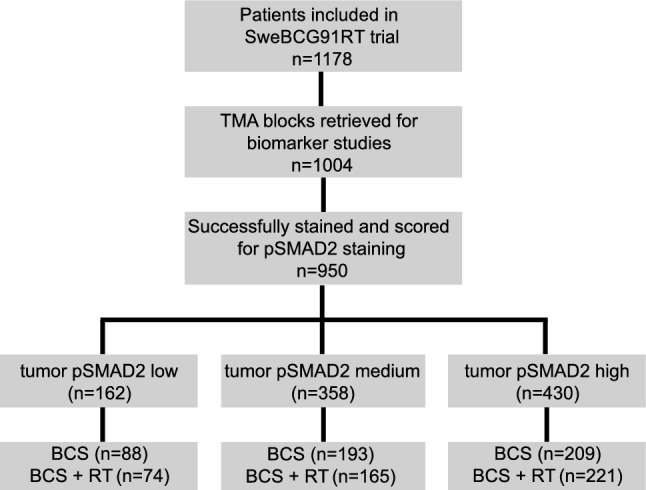
CONSORT diagram. Patients from the Swedish Breast Cancer Group 91 Radiotherapy (SweBCG91RT) randomized radiotherapy trial included in the present study. TMA tissue microarray, BCS breast cancer surgery, RT radiotherapy

Tissue cores of 950 patients were successfully stained and annotated for the fraction of positive nuclear pSMAD2 in both tumor and stroma cell compartments (CONSORT diagram, Fig. 1). The score distribution revealed a comparable left-skewed distribution pattern toward high fractions of positive SMAD2 staining for both compartments (Supplementary Fig. 2A). Accordingly, a strong positive correlation was observed between the fraction of positive tumoral and stromal pSMAD2 staining (*p* < 0.001, Spearman’s Rank test; Supplementary Fig. 2B). Consequently, we decided to focus on the tumor cell compartment in the following analyses on clinical associations.

Guided by the distribution of the tumoral pSMAD2 scores, patients were categorized into tumoral pSMAD2 low (≤ 20% positive cells; *n* = 162), medium (21–79%; *n* = 358), and high (≥ 80%; *n* = 430) status groups (Supplementary Fig. 2A).

### Associations between tumoral pSMAD2 status and clinicopathological patient characteristics

The clinicopathological characteristics of the study cohort are outlined in Table 1. Tumors with low pSMAD2 levels tended to have larger size compared to those with medium or high pSMAD2 levels (median tumor size 15 mm vs. 12 mm and 12 mm, respectively; *p* < 0.001, ANOVA test). Furthermore, lower pSMAD2 level was associated with a higher likelihood of grade III tumors (*p* < 0.001, Pearson’s chi-squared test). No significant associations were observed between tumoral pSMAD2 levels and age, HER2 and hormone receptor group, or radiotherapy group (Table 1).

**Table 1:** Associations between tumoral pSMAD2 status and clinicopathologic patient characteristics, as well as immune markers

	pSmad2 tumor				*p *Value
	Low (0.5–20%)	Medium (21–79%)	High (80–100%)	Total	
	*N* = 162	*N* = 358	*N* = 430	*N* = 950	
Age, Median (IQR)	59.0 (52.0–66.0)	59.0 (50.0–66.0)	60.0 (52.0–67.0)	59.0 (51.0–66.0)	0.20
Tumor size (mm), median (IQR)	15.0 (10.0–19.0)	12.0 (10.0–15.0)	12.0 (9.0–15.0)	12.0 (10.0–16.0)	** < 0.001**
Receptor group					0.39
Luminal A	87 (55.8%)	206 (59.4%)	233 (56.3%)	526 (57.4%)	
Luminal B	38 (24.4%)	92 (26.5%)	121 (29.2%)	251 (27.4%)	
HER2/Triple neg	31 (19.9%)	49 (14.1%)	60 (14.5%)	140 (15.3%)	
HER2 group					0.80
0	69 (43.7%)	167 (47.9%)	200 (48.2%)	436 (47.3%)	
1+/2+	75 (47.5%)	159 (45.6%)	188 (45.3%)	422 (45.8%)	
3+/ampl	14 (8.9%)	23 (6.6%)	27 (6.5%)	64 (6.9%)	
Histologic grade					** < 0.001**
Grade 1	21 (13.5%)	56 (16.8%)	61 (15.4%)	138 (15.6%)	
Grade 2	74 (47.7%)	191 (57.2%)	264 (66.5%)	529 (59.7%)	
Grade 3	60 (38.7%)	87 (26.0%)	72 (18.1%)	219 (24.7%)	
RT					0.25
No	88 (54.3%)	193 (53.9%)	209 (48.6%)	490 (51.6%)	
yes	74 (45.7%)	165 (46.1%)	221 (51.4%)	460 (48.4%)	
TILs					0.065
< 10%	102 (64.6%)	241 (69.3%)	308 (74.0%)	651 (70.6%)	
> 10%	56 (35.4%)	107 (30.7%)	108 (26.0%)	271 (29.4%)	
CD8					0.35
0–9%	10 (6.2%)	18 (5.0%)	20 (4.7%)	48 (5.1%)	
10–49%	56 (34.8%)	148 (41.3%)	188 (44.2%)	392 (41.5%)	
50–100%	95 (59.0%)	192 (53.6%)	217 (51.1%)	504 (53.4%)	
FOXP3					**0.020**
0	22 (13.7%)	30 (8.4%)	34 (8.0%)	86 (9.1%)	
1–9%	106 (65.8%)	246 (68.7%)	323 (76.0%)	675 (71.5%)	
10–74%	33 (20.5%)	82 (22.9%)	68 (16.0%)	183 (19.4%)	
PD1					0.68
< 1%	129 (87.2%)	265 (84.9%)	298 (87.1%)	692 (86.3%)	
≥1%	19 (12.8%)	47 (15.1%)	44 (12.9%)	110 (13.7%)	
PDL1					0.64
< 1%	115 (77.7%)	248 (79.5%)	278 (81.3%)	641 (79.9%)	
≥ 1%	33 (22.3%)	64 (20.5%)	64 (18.7%)	161 (20.1%)	

Data are presented as median for continuous measures, and *n* (%) for categorical measures

*IQR* interquartile range, *TILs* tumor-infiltrating lymphocytes, *RT* radiotherapy

For continuous variables pSMAD2 groups are compared using Anova test, and for categorical variables groups are compared using Pearson’s chi-squared test; *p* values < 0.05 in bold text

### Prognostic impact of the tumoral pSMAD2 status on IBTR

**Fig. 2:**
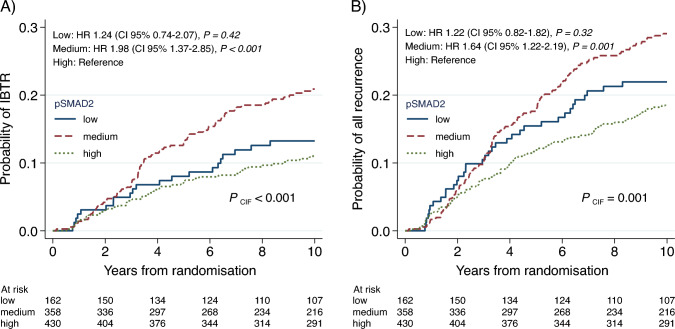
Prognostic impact of the tumoral pSMAD2 status. Cumulative incidence of **A** IBTR and **B** all types of recurrence combined, across the different tumoral pSMAD2 status groups. The dotted green line represents the high pSMAD2 status group, the dashed red line represents the medium status group, and the blue line represents the low status group. *P*_CIF_ value is based on the cumulative incidence function. Additionally, univariable Cox regression analysis of relative risk is provided, with the pSMAD2 high group as reference and corresponding *p *values based on Wald test. IBTR ipsilateral breast tumor recurrence, HR Hazard ratio, CI confidence interval, CIF cumulative incidence function

Patients with medium tumoral pSMAD2 status exhibited a significantly higher cumulative incidence and increased risk of IBTR in univariable Cox regression analysis compared to the reference patient group with high pSMAD2 status (*P*_CIF_ < 0.001; HR 1.98, CI 95% 1.37–2.85, *p* < 0.001) (Fig. 2A, Table 2). However, patients in the low pSMAD2 status group did not show a significantly different IBTR risk than those in the high group (HR_uni_ 1.24, CI 95% 0.74–2.07, *p* = 0.42) (Fig. 2A, Table 2). In a multivariable analysis including histological grade, age, and RT, the significance remained and distinguished the pSMAD2 medium group (HR_multi_ 1.82 CI 95% (1.24–2.65), *p* = 0.002) (Table 2).

**Table 2:** Cox proportional hazard regression for IBTR, 10-year follow-up

	Number of events/Number of patients (%)	Univariable Cox regression HR (CI 95%)	*p *Value	Multivariable Cox regression HR (CI 95%)	*p *Value
pSMAD2 tumor					
0.5–20%	21/162 (13.0%)	1.24 (0.74–2.07)	0.42	1.09 (0.64–1.87)	0.75
21–79%	74/358 (20.7%)	1.98 (1.37–2.85)	< 0.001	1.82 (1.24–2.65)	**0.002**
80–100%	47/430 (10.9%)	Ref		Ref	
Radiotherapy					
No	103/490 (21.0%)	Ref.^a^		Ref.^a^	
Yes	39/460 (8.5%)	0.36 (0.25–0.53)	< 0.001	0.39 (0.27–0.57)	** < 0.001**
Age					
Years, continuous	142/950 (15.0%)	0.97 (0.96–0.99)	0.003	0.98 (0.96–0.99)	**0.005**
Tumor size					
cm, continuous	144/944 (14.8%)	0.89 (0.65–1.22)	0.49		
Histology grade					
1	13/133 (9.8%)	Ref		Ref	
2	82/549 (14.9%)	1.65 (0.92–2.97)	0.093	1.69 (0.94–3.05)	0.078
3	48/232 (17.2%)	2.13 (1.14–3.98)	0.018	1.91 (1.02–3.59)	**0.042**
Missing	7/36 (19.4%)				
Receptor group					
Luminal A	73/526 (13.9%)	Ref			
Luminal B	39/251 (15.5%)	1.20 (0.81–1.77)	0.36		
HER2/triple neg	22/140(15.7%)	1.29 (0.80–2.07)	0.30		
Missing	8/33 (24.2%)				

*IBTR* ipsilateral breast tumor recurrence, *HR* Hazard ratio, *CI* confidence interval, *RT* radiotherapy

*p* values are based on Wald test; *p* values < 0.05 in bold text. Only variables significant in univariable analysis were included in the multivariable regression model

^a^The proportional hazards assumption was not met, HR should thus be interpreted as average over time

Similarly, for the endpoint combining all recurrence types, only patients with medium tumoral pSMAD2 status showed a significantly increased cumulative incidence and risk compared to the pSMAD2 high reference group (Fig. 2B, Supplementary Table 1).

### Impact of the tumoral pSMAD2 status on benefit from radiotherapy

**Fig. 3:**
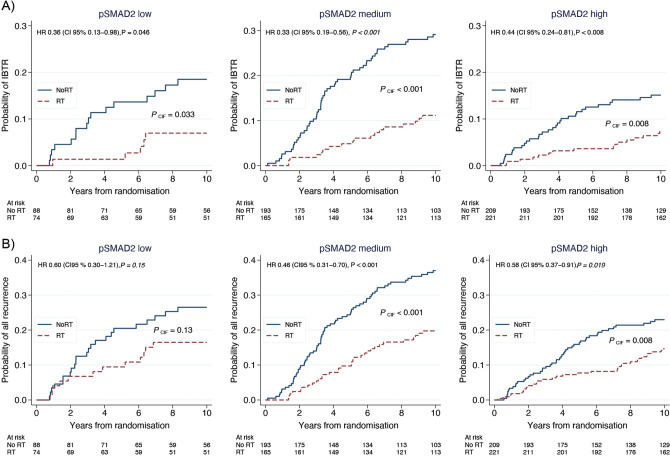
Impact of the tumoral pSMAD2 status on the benefit of radiotherapy. Cumulative incidence with or without adjuvant RT of **A** IBTR and **B** all types of recurrence combined, across patients within the different tumoral pSMAD2 status groups. Blue lines represent patients not receiving adjuvant RT treatment (noRT) while red dashed lines represent those receiving adjuvant RT treatment (RT).* P*_CIF_ value is based on the cumulative incidence function. Additionally, univariable Cox regression analysis of relative risk is provided, with no RT patients serving as reference and corresponding *p *values based on Wald test. IBTR ipsilateral breast tumor recurrence, HR hazard ratio, CI confidence interval, CIF cumulative incidence function, RT radiotherapy

The risk reduction in IBTR associated with RT was consistently significant across all tumoral pSMAD2 patient groups: high (HR 0.44, 95% CI 0.24–0.81, *p* = 0.008), medium (HR 0.33, 95% CI 0.19–0.56, *p* < 0.001), and low (HR 0.36, 95% CI 0.13–0.98, *p* = 0.046) (Fig. 3A). Consequently, no significant interaction was observed between the pSMAD2 status group and RT (*p*_interaction_ = 0.79).

Similarly, a notable risk reduction by RT for all recurrence types combined was observed across all three pSMAD2 patient groups. However, the effect reached statistical significance only in the high and medium pSMAD2 groups, while the low pSMAD2 group showed a strong tendency (Fig. 3B).

### Association of tumoral pSMAD2 and immune status

To evaluate the potential impact of TGF-β signaling on the tumor immune status, we correlated the tumoral pSMAD2 groups with tumor-infiltrating lymphocytes (TILs), the lymphocyte markers CD8 and FOXP3, as well as the immune checkpoint markers PD1 and PDL1 using categorized variables. We observed a tendency suggesting a negative association between tumoral pSMAD2 status and the presence of TILs (*p* = 0.065, chi-squared test). Additionally, there was a negative association between tumoral pSMAD2 status and FOXP3-positive cells (*p* = 0.020, chi-squared test) (Table 1). However, no associations were noted between the pSMAD2 groups and CD8, PD1, or PDL1.

**Fig. 4:**
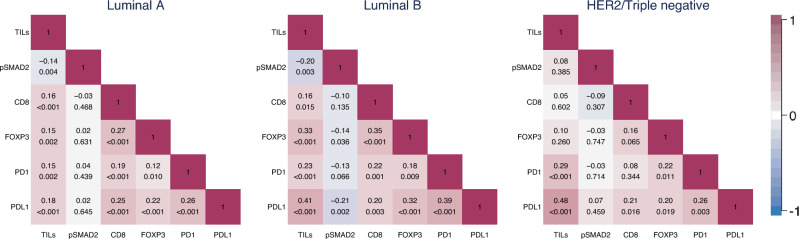
Correlation of tumoral pSMAD2 level with lymphocytes and PD1/PDL1 immune checkpoint proteins separated by breast cancer subtype. The numbers in the rectangles are Spearman’s rank correlation coefficients and *p* values for the compared variables. TILs tumor-infiltrating lymphocytes

Given the varying clinical implications and prevalence of TILs across breast cancer subtypes [42], we performed a refined correlation analysis separately for Luminal A, Luminal B, and Her2+/Triple-negative patients using continuous immune marker scores. Tumoral pSMAD2 and the overall TILs score revealed a significant, though weak negative correlation in Luminal A and B subtypes only (*ρ* = − 0.14, *p* = 0.004, and *ρ* = − 0.20, *p* = 0.003, Spearman Rank test). In addition, Luminal B cases also showed a significant negative correlation between tumoral pSMAD2 and FOXP3 (*ρ* = − 0.14, *p* = 0.036) as well as tumoral pSMAD2 and PD-L1 (*ρ* = − 0.21, *p* = 0.002) (Fig. 4). No significant associations with immune markers and tumoral pSMAD2 status were detected for the Her2+/Triple-negative patient group.

## Discussion

In this study, we investigated the role of TGF-β in a well-defined cohort of early-stage, node-negative invasive breast cancer patients who were part of the SweBCG91RT randomized radiotherapy trial. We used phosphorylated SMAD2 as a surrogate marker to assess active TGF-β signaling. TGF-β is known to have both tumor-promoting as well as restraining functions, depending on tumor type, tumor characteristics as well as the status of the tumor microenvironment [10–13]. The role of TGF-β in breast cancer is therefore still not fully understood, posing challenges to the implementation of TGF-β targeting therapies, despite available and specific inhibitors such as galunisertib or fresolimumab [22].

We found that 45% of early-stage breast cancer patients exhibited high pSMAD2 levels, defined as having ≥ 80% of positive tumor cells. Interestingly, patients with medium pSMAD2 had a significantly increased IBTR as well as any recurrence risk compared to the pSMAD2 high group.

These results may suggest that, in early-stage breast cancers, TGF-β signaling still exhibits its tumor-suppressive, anti-mitogenic function, thereby limiting progression and lowering recurrence risk. Preclinical studies suggest, that during tumor progression, key components of the canonical TGF-β pathway (e.g., SMAD2/3, SMAD4, TGFBR1/2) are often epigenetically downregulated via histone modifications, potentially leading to non-canonical pathway activation with pro-tumorigenic signaling routes [46, 47]. Consistent with this, we found that tumors with medium or low nuclear pSMAD2 were larger and of higher grade than those with high pSMAD2. Importantly, even after adjusting for standard clinicopathological factors, medium pSMAD2 remained an independent predictor of increased recurrence risk, indicating that attenuation of TGF-β signaling may serve as a prognostic marker at early breast cancer stages. Of note though, no significant differences were noted between the pSMAD2 low group and the medium or high groups with regard to IBTR or overall recurrence risk. This patient group may represent a distinct subset with unique downstream signaling. However, since pSMAD2 low patients comprise only 17% of our cohort, this finding should be interpreted cautiously.

Future studies incorporating mutational and expression data on TGF-β pathway components are needed to improve patient stratification and therapeutic targeting. Unfortunately, in our early-stage breast cancer cohort, no information was available regarding the mutational status or gene expression level of TGF-β signaling components. The value of integrating TGF-β downstream signaling elements in breast cancer biomarker studies was highlighted in a study on invasive breast cancer, which included a substantial number of node-positive patients [38]. This study demonstrated that protein levels of the tumor suppressor SMAD4 could further stratify the pSMAD2 high patient group, revealing significantly reduced relapse-free survival rates for pSMAD2 high/SMAD4 low patients compared to pSMAD2 high/SMAD4 high patients. This effect may be attributed to the onset of non-canonical TGF-β signaling in the absence of SMAD4, impairing the anti-proliferative action of TGF-β [48]. Our future efforts should consider to integrate SMAD4 into our pSMAD2 analyses, ideally, by investigating their direct interaction.

Based on our findings, targeting TGF-β in early-stage breast cancer patients without appropriate biomarker guidance may be unfavorable. While our data suggests pSMAD2 as a potential biomarker, we acknowledge the technical challenges and limitations associated with our study. Tumoral nuclear pSMAD2 positivity was scored by a board-certified pathologist specializing in breast cancer pathology using a predefined staining cut-off designed for clinical relevance. Future efforts should focus on developing a standardized scoring system for pSMAD2 IHC. pSMAD2 may transiently localize in the cytoplasm, and consequently, in numerous cases a significant background cytoplasmic staining was observed, complicating the interpretation of nuclear staining. Digital approaches are essential to standardize and ensure the transferability of pSMAD2 assessment, but despite significant advancements in AI-based tissue annotation tools, the accurate digital discrimination between tumor and stroma cells based solely on hematoxylin staining remains problematic. Multiplex fluorescent approaches, incorporating specific markers for tumor and stroma cells, could enhance the digital assessment of tumoral pSMAD2 and should be considered; yet, as for now, multiplex approaches are difficult to integrate into routine clinical diagnostics. Another important limitation of our study is the low number of HER2+ and triple-negative cases, which prevents meaningful subtype-specific analyses. That said, the subtype distribution of our randomized trial cohort reflects reported distribution of early-stage breast cancer [49, 50], and we observed no statistically significant difference in pSMAD2 levels between subtypes (Table 1). Given the generally favorable prognosis and low recurrence rates in early-stage breast cancers, we decided not to split the cohort into the individual subtypes for recurrence risk analyses to preserve statistical power. Consequently, our findings are driven predominantly by Luminal tumors (84.8%) and offer very limited insight into early-stage HER2+ and triple-negative breast cancers. This gap warrants dedicated studies in these subgroups, especially since analyses of later-stage disease have revealed subtype-specific effects, such as HER2-driven SMAD3 phosphorylation, and our group and others have demonstrated that the tumor microenvironment carries distinct molecular characteristics and clinical value in the different subtypes [42, 51, 52].

Indeed, our analysis showed that the inverse relationship between tumoral pSMAD2 levels and overall TILs density was confined to the Luminal subtypes and was not present in the pooled HER2+/triple-negative patients. Furthermore, only Luminal B tumors displayed that higher pSMAD2 correlate with reduced FOXP3 and PD-L1 expression. Our earlier work on the same cohort demonstrated that total TILs numbers, and thereby also specific subsets including CD8⁺ and FOXP3⁺ cells, rise progressively from Luminal A through Luminal B to HER2⁺ and Triple-negative subtypes [42, 43]. Such subtype-specific immune landscapes imply that TGF-β signaling also exerts its immunomodulatory effects in concert with tumor intrinsic pathways, leading to differential immune activation or suppression across subtypes. This again highlights the context-specific nature of TGF-β signaling and underscores the importance of well-defined patient groups for determining the optimal therapeutical setting.

The immunomodulatory effects of TGF-β not only indirectly affect tumor progression but also treatment responses. RT generates “danger” signals in tissues that, under certain conditions, enhance tumor antigen presentation and thus anti-cancer immunity [14, 53]. A parallel inhibition of TGF-β signaling during RT is therefore expected to enhance RT efficacy by promoting immune cell activation [54]. Indeed, findings from a randomized trial in metastatic breast cancer, where patients receiving a high dose of the TGF-β1,2,3 neutralizing antibody fresolimumab experienced prolonged overall survival compared to the lower dose group, support a role of TGF-β signaling in RT response [55]. Our retrospective analysis focused on lymph node-negative, early-stage breast cancer patients does not indicate that the increased IBTR or any recurrence risk observed in the pSMAD2 medium patient group is due to differential effects of adjuvant RT based on TGF-β signaling in the primary tumor, as proven by the consistent and significant efficacy of RT across all pSMAD2 patient groups, with a non-significant formal interaction test between pSMAD2 group and RT.

In conclusion, using nuclear pSMAD2 as a marker for active TGF-β signaling in a large cohort of early-stage breast cancer patients, our findings suggest that TGF-β signaling may play an anti-tumoral role by reducing recurrence risk, challenging its therapeutic targeting in this patient group. We observed no interaction between TGF-β status and benefit from adjuvant radiotherapy. In Luminal subtypes, higher pSMAD2 correlated inversely with TIL density, highlighting subtype-specific immunomodulatory effects. Moving forward, it is crucial to standardize pSMAD2 assessment methods and to expand analyses to larger HER2+ and Triple-negative cohorts of early-stage breast cancer to fully decipher subtype-specific roles of TGF-β signaling.

## Supplementary Information

Below is the link to the electronic supplementary material.

Supplementary file1 (PDF 3224 KB)

## Data Availability

The clinical data table will be provided upon request.

## References

[1] Massagué J, Sheppard D (2023) TGF-β signaling in health and disease. Cell 186:4007–4037. 10.1016/j.cell.2023.07.036 37714133 10.1016/j.cell.2023.07.036 PMC10772989

[2] Padua D, Zhang XH-F, Wang Q et al (2008) TGFbeta primes breast tumors for lung metastasis seeding through angiopoietin-like 4. Cell 133:66–77. 10.1016/j.cell.2008.01.046 18394990 10.1016/j.cell.2008.01.046 PMC2390892

[3] de Graauw M, van Miltenburg MH, Schmidt MK et al (2010) Annexin A1 regulates TGF-β signaling and promotes metastasis formation of basal-like breast cancer cells. Proc Natl Acad Sci USA 107:6340–6345. 10.1073/pnas.0913360107 20308542 10.1073/pnas.0913360107 PMC2852023

[4] Hu M, Yao J, Carroll DK et al (2008) Regulation of in situ to invasive breast carcinoma transition. Cancer Cell 13:394–406. 10.1016/j.ccr.2008.03.007 18455123 10.1016/j.ccr.2008.03.007 PMC3705908

[5] Hanks BA, Holtzhausen A, Evans KS et al (2013) Type III TGF-β receptor downregulation generates an immunotolerant tumor microenvironment. J Clin Invest 123:3925–3940. 10.1172/JCI65745 23925295 10.1172/JCI65745 PMC3754240

[6] Drabsch Y, ten Dijke P (2011) TGF-β signaling in breast cancer cell invasion and bone metastasis. J Mammary Gland Biol Neoplasia 16:97–108. 10.1007/s10911-011-9217-1 21494783 10.1007/s10911-011-9217-1 PMC3095797

[7] Batlle E, Massagué J (2019) Transforming growth factor-β signaling in immunity and cancer. Immunity 50:924–940. 10.1016/j.immuni.2019.03.024 30995507 10.1016/j.immuni.2019.03.024 PMC7507121

[8] Heldin C-H, Moustakas A (2016) Signaling receptors for TGF-β family members. Cold Spring Harb Perspect Biol 8:a022053. 10.1101/cshperspect.a022053 27481709 10.1101/cshperspect.a022053 PMC4968163

[9] Sundqvist A, ten Dijke P, van Dam H (2012) Key signaling nodes in mammary gland development and cancer: Smad signal integration in epithelial cell plasticity. Breast Cancer Res 14:204. 10.1186/bcr3066 22315972 10.1186/bcr3066 PMC3496114

[10] David CJ, Massagué J (2018) Contextual determinants of TGFβ action in development, immunity and cancer. Nat Rev Mol Cell Biol 19:419–435. 10.1038/s41580-018-0007-0 29643418 10.1038/s41580-018-0007-0 PMC7457231

[11] Derynck R, Akhurst RJ, Balmain A (2001) TGF-beta signaling in tumor suppression and cancer progression. Nat Genet 29:117–129. 10.1038/ng1001-117 11586292 10.1038/ng1001-117

[12] Bierie B, Moses HL (2006) Tumour microenvironment: TGFbeta: the molecular Jekyll and Hyde of cancer. Nat Rev Cancer 6:506–520. 10.1038/nrc1926 16794634 10.1038/nrc1926

[13] Seoane J, Gomis RR (2017) TGF-β family signaling in tumor suppression and cancer progression. Cold Spring Harb Perspect Biol 9:a022277. 10.1101/cshperspect.a022277 28246180 10.1101/cshperspect.a022277 PMC5710110

[14] Liao R-Y, Mao C, Qiu L-X et al (2010) TGFBR1*6A/9A polymorphism and cancer risk: a meta-analysis of 13,662 cases and 14,147 controls. Mol Biol Rep 37:3227–3232. 10.1007/s11033-009-9906-7 19882361 10.1007/s11033-009-9906-7

[15] Lin L-H, Chang K-W, Cheng H-W, Liu C-J (2019) SMAD4 somatic mutations in head and neck carcinoma are associated with tumor progression. Front Oncol 9:1379. 10.3389/fonc.2019.01379 31867281 10.3389/fonc.2019.01379 PMC6909744

[16] Hancock BA, Chen Y-H, Solzak JP et al (2019) Profiling molecular regulators of recurrence in chemorefractory triple-negative breast cancers. Breast Cancer Res 21:87. 10.1186/s13058-019-1171-7 31383035 10.1186/s13058-019-1171-7 PMC6683504

[17] Rong L, Li R, Li S, Luo R (2016) Immunosuppression of breast cancer cells mediated by transforming growth factor-β in exosomes from cancer cells. Oncol Lett 11:500–504. 10.3892/ol.2015.3841 26870240 10.3892/ol.2015.3841 PMC4727188

[18] Mariathasan S, Turley SJ, Nickles D et al (2018) TGFβ attenuates tumour response to PD-L1 blockade by contributing to exclusion of T cells. Nature 554:544–548. 10.1038/nature25501 29443960 10.1038/nature25501 PMC6028240

[19] Ghiringhelli F, Ménard C, Terme M et al (2005) CD4+CD25+ regulatory T cells inhibit natural killer cell functions in a transforming growth factor-beta-dependent manner. J Exp Med 202:1075–1085. 10.1084/jem.20051511 16230475 10.1084/jem.20051511 PMC2213209

[20] Tauriello DVF, Palomo-Ponce S, Stork D et al (2018) TGFβ drives immune evasion in genetically reconstituted colon cancer metastasis. Nature 554:538–543. 10.1038/nature25492 29443964 10.1038/nature25492

[21] Chakravarthy A, Khan L, Bensler NP et al (2018) TGF-β-associated extracellular matrix genes link cancer-associated fibroblasts to immune evasion and immunotherapy failure. Nat Commun 9:4692. 10.1038/s41467-018-06654-8 30410077 10.1038/s41467-018-06654-8 PMC6224529

[22] Ciardiello D, Elez E, Tabernero J, Seoane J (2020) Clinical development of therapies targeting TGFβ: current knowledge and future perspectives. Ann Oncol 31:1336–1349. 10.1016/j.annonc.2020.07.009 32710930 10.1016/j.annonc.2020.07.009

[23] Ganapathy V, Ge R, Grazioli A et al (2010) Targeting the transforming growth factor-beta pathway inhibits human basal-like breast cancer metastasis. Mol Cancer 9:122. 10.1186/1476-4598-9-122 20504320 10.1186/1476-4598-9-122 PMC2890606

[24] Rausch MP, Hahn T, Ramanathapuram L et al (2009) An orally active small molecule TGF-beta receptor I antagonist inhibits the growth of metastatic murine breast cancer. Anticancer Res 29:2099–2109 19528470 PMC2860108

[25] Zhang M, Kleber S, Röhrich M et al (2011) Blockade of TGF-β signaling by the TGFβR-I kinase inhibitor LY2109761 enhances radiation response and prolongs survival in glioblastoma. Cancer Res 71:7155–7167. 10.1158/0008-5472.CAN-11-1212 22006998 10.1158/0008-5472.CAN-11-1212

[26] Biswas S, Nyman JS, Alvarez J et al (2011) Anti-transforming growth factor ß antibody treatment rescues bone loss and prevents breast cancer metastasis to bone. PLoS ONE 6:e27090. 10.1371/journal.pone.0027090 22096521 10.1371/journal.pone.0027090 PMC3214031

[27] Zhong Z, Carroll KD, Policarpio D et al (2010) Anti-transforming growth factor beta receptor II antibody has therapeutic efficacy against primary tumor growth and metastasis through multieffects on cancer, stroma, and immune cells. Clin Cancer Res 16:1191–1205. 10.1158/1078-0432.CCR-09-1634 20145179 10.1158/1078-0432.CCR-09-1634

[28] Grenga I, Donahue RN, Gargulak ML et al (2018) Anti-PD-L1/TGFβR2 (M7824) fusion protein induces immunogenic modulation of human urothelial carcinoma cell lines, rendering them more susceptible to immune-mediated recognition and lysis. Urol Oncol 36:93.e1–e93.e11. 10.1016/j.urolonc.2017.09.027 29103968 10.1016/j.urolonc.2017.09.027 PMC5835162

[29] Ravi R, Noonan KA, Pham V et al (2018) Bifunctional immune checkpoint-targeted antibody-ligand traps that simultaneously disable TGFβ enhance the efficacy of cancer immunotherapy. Nat Commun 9:741. 10.1038/s41467-017-02696-6 29467463 10.1038/s41467-017-02696-6 PMC5821872

[30] Muraoka RS, Dumont N, Ritter CA et al (2002) Blockade of TGF-beta inhibits mammary tumor cell viability, migration, and metastases. J Clin Invest 109:1551–1559. 10.1172/JCI15234 12070302 10.1172/JCI15234 PMC151012

[31] Teixeira AF, Ten Dijke P, Zhu H-J (2020) On-target anti-TGF-β therapies are not succeeding in clinical cancer treatments: what are remaining challenges? Front Cell Dev Biol 8:605. 10.3389/fcell.2020.00605 32733895 10.3389/fcell.2020.00605 PMC7360684

[32] Sato M, Kadota M, Tang B et al (2014) An integrated genomic approach identifies persistent tumor suppressive effects of transforming growth factor-β in human breast cancer. Breast Cancer Res 16:R57. 10.1186/bcr3668 24890385 10.1186/bcr3668 PMC4095608

[33] Yang Y, Yang HH, Tang B et al (2020) The outcome of TGFβ antagonism in metastatic breast cancer models in vivo reflects a complex balance between tumor-suppressive and proprogression activities of TGFβ. Clin Cancer Res 26:643–656. 10.1158/1078-0432.CCR-19-2370 31582516 10.1158/1078-0432.CCR-19-2370 PMC8182485

[34] Walker RA, Dearing SJ, Gallacher B (1994) Relationship of transforming growth factor beta 1 to extracellular matrix and stromal infiltrates in invasive breast carcinoma. Br J Cancer 69:1160–1165. 10.1038/bjc.1994.228 7515264 10.1038/bjc.1994.228 PMC1969439

[35] Desruisseau S, Palmari J, Giusti C et al (2006) Determination of TGFbeta1 protein level in human primary breast cancers and its relationship with survival. Br J Cancer 94:239–246. 10.1038/sj.bjc.6602920 16404434 10.1038/sj.bjc.6602920 PMC2361106

[36] Figueroa JD, Flanders KC, Garcia-Closas M et al (2010) Expression of TGF-beta signaling factors in invasive breast cancers: relationships with age at diagnosis and tumor characteristics. Breast Cancer Res Treat 121:727–735. 10.1007/s10549-009-0590-z 19937272 10.1007/s10549-009-0590-z PMC4159718

[37] Paiva CE, Drigo SA, Rosa FE et al (2010) Absence of transforming growth factor-beta type II receptor is associated with poorer prognosis in HER2-negative breast tumours. Ann Oncol 21:734–740. 10.1093/annonc/mdp518 19914962 10.1093/annonc/mdp518

[38] de Kruijf EM, Dekker TJA, Hawinkels LJAC et al (2013) The prognostic role of TGF-β signaling pathway in breast cancer patients. Ann Oncol 24:384–390. 10.1093/annonc/mds333 23022998 10.1093/annonc/mds333

[39] Gil Del Alcazar CR, Huh SJ, Ekram MB et al (2017) Immune escape in breast cancer during in situ to invasive carcinoma transition. Cancer Discov 7:1098–1115. 10.1158/2159-8290.CD-17-0222 28652380 10.1158/2159-8290.CD-17-0222 PMC5628128

[40] Malmström P, Holmberg L, Anderson H et al (2003) Breast conservation surgery, with and without radiotherapy, in women with lymph node-negative breast cancer: a randomised clinical trial in a population with access to public mammography screening. Eur J Cancer 39:1690–1697. 10.1016/s0959-8049(03)00324-1 12888363 10.1016/s0959-8049(03)00324-1

[41] Sjöström M, Lundstedt D, Hartman L et al (2017) Response to radiotherapy after breast-conserving surgery in different breast cancer subtypes in the Swedish Breast Cancer Group 91 radiotherapy randomized clinical trial. J Clin Oncol 35:3222–3229. 10.1200/JCO.2017.72.7263 28759347 10.1200/JCO.2017.72.7263

[42] Kovács A, Stenmark Tullberg A, Werner Rönnerman E et al (2019) Effect of radiotherapy after breast-conserving surgery depending on the presence of tumor-infiltrating lymphocytes: a long-term follow-up of the SweBCG91RT randomized trial. J Clin Oncol 37:1179–1187. 10.1200/JCO.18.02157 30939091 10.1200/JCO.18.02157

[43] Stenmark Tullberg A, Puttonen HAJ, Sjöström M et al (2021) Immune infiltrate in the primary tumor predicts effect of adjuvant radiotherapy in breast cancer; results from the randomized SweBCG91RT trial. Clin Cancer Res 27:749–758. 10.1158/1078-0432.CCR-20-3299 33148672 10.1158/1078-0432.CCR-20-3299

[44] Stenmark Tullberg A, Sjöström M, Tran L et al (2023) Combining histological grade, TILs, and the PD-1/PD-L1 pathway to identify immunogenic tumors and de-escalate radiotherapy in early breast cancer: a secondary analysis of a randomized clinical trial. J Immunother Cancer 11:e006618. 10.1136/jitc-2022-006618 37208129 10.1136/jitc-2022-006618 PMC10201214

[45] Fine JP, Gray RJ (1999) A proportional hazards model for the subdistribution of a competing risk. J Am Stat Assoc 94:496–509. 10.1080/01621459.1999.10474144

[46] Hinshelwood RA, Huschtscha LI, Melki J et al (2007) Concordant epigenetic silencing of transforming growth factor-β signaling pathway genes occurs early in breast carcinogenesis. Can Res 67:11517–11527. 10.1158/0008-5472.CAN-07-1284 10.1158/0008-5472.CAN-07-1284 18089780

[47] Zhang YE (2009) Non-Smad pathways in TGF-β signaling. Cell Res 19:128–139. 10.1038/cr.2008.328 19114990 10.1038/cr.2008.328 PMC2635127

[48] Deckers M, van Dinther M, Buijs J et al (2006) The tumor suppressor Smad4 is required for transforming growth factor beta-induced epithelial to mesenchymal transition and bone metastasis of breast cancer cells. Cancer Res 66:2202–2209. 10.1158/0008-5472.CAN-05-3560 16489022 10.1158/0008-5472.CAN-05-3560

[49] NKBC. https://statistik.incanet.se/brostcancer/. Accessed 26 Apr 2025

[50] Kong X, Liu Z, Cheng R et al (2020) Variation in breast cancer subtype incidence and distribution by race/ethnicity in the United States from 2010 to 2015. JAMA Netw Open 3:e2020303. 10.1001/jamanetworkopen.2020.20303 33074325 10.1001/jamanetworkopen.2020.20303 PMC7573683

[51] Lesurf R, Aure MR, Mørk HH et al (2016) Molecular features of subtype-specific progression from ductal carcinoma in situ to invasive breast cancer. Cell Rep 16:1166–1179. 10.1016/j.celrep.2016.06.051 27396337 10.1016/j.celrep.2016.06.051

[52] Hu M, Yao J, Cai L et al (2005) Distinct epigenetic changes in the stromal cells of breast cancers. Nat Genet 37:899–905. 10.1038/ng1596 16007089 10.1038/ng1596

[53] McBride WH, Chiang C-S, Olson JL et al (2004) A sense of danger from radiation. Radiat Res 162:1–19. 10.1667/rr3196 15222781 10.1667/rr3196

[54] Demaria S, Bhardwaj N, McBride WH, Formenti SC (2005) Combining radiotherapy and immunotherapy: a revived partnership. Int J Radiat Oncol Biol Phys 63:655–666. 10.1016/j.ijrobp.2005.06.032 16199306 10.1016/j.ijrobp.2005.06.032 PMC1489884

[55] Formenti SC, Lee P, Adams S et al (2018) Focal irradiation and systemic TGFβ blockade in metastatic breast cancer. Clin Cancer Res 24:2493–2504. 10.1158/1078-0432.CCR-17-3322 29476019 10.1158/1078-0432.CCR-17-3322 PMC5999326

