# Supplementary material for: Tumoral pSMAD2 as a prognostic biomarker in early-stage breast cancer: insights from the randomized SweBCG91RT trial
Source: Breast Cancer Res Treat. 2025 Jun 9;212(3):499–509. doi: 10.1007/s10549-025-07744-0 (PMC12208964; doi:10.1007/s10549-025-07744-0)
Supplement: Supplementary file 1 — Supplementary file1 (PDF 3224 KB) [file 10549_2025_7744_MOESM1_ESM.pdf]

Supplementary material

Tumoral pSMAD2 as a Prognostic Biomarker in Early-Stage Breast Cancer: Insights from the Randomized SweBCG91RT Trial

Axel Stenmark Tullberg, Viktoria Thurfjell, Anikó Kovács, Patrick Micke, Aristidis Moustakas, Fredrika Killander, Emma Niméus, Erik Holmberg, Per Karlsson, Carina Strell

Page 2 **Supplementary Table 1**
”Cox proportional hazard regression for all recurrence”

Page 3 **Supplementary Figure 1**
“pSMAD2 IHC staining controls and staining examples”

Page 4 **Supplementary Figure 2**
”pSMAD2 staining score distribution”

	Number of events/ Number of patients (%)	Univariable Cox regression HR (CI 95%)	*P*	Multivariable Cox regression HR (CI 95%)	*P*
**pSMAD2 tumor**					
0.5-20%	35/162 (21.6%)	1.22 (0.82-1.82)	0.321	1.12 (0.74-1.70)	0.600
21-79%	103/358 (28.8%)	1.64 (1.22-2.19)	0.001	1.58 (1.16-2.15)	**0.003**
80-100%	79/430 (18.4%)	Ref.		Ref.	
**Radiotherapy**					
No	141/490 (28.8%)	Ref.*		Ref.*	
Yes	76/460 (16.5%)	0.52 (0.39-0.69)	<0.001	0.58 (0.44-0.78)	**<0.001**
**Age**					
Years, continuous	217/950 (22.8%)	0.98 (0.96-0.99)	0.001	0.98 (0.96-0.99)	**0.001**
**Tumor size**					
cm, continuous	215/944(22.8%)	1.34 (1.08-1.68)	0.009		
**Histology grade**					
1	17/133 (12.8%)	Ref.*		Ref.	
2	118/549 (21.5%)	1.82 (1.09-3.02)	0.021	1.80 (1.08-3.00)	**0.025**
3	73/232 (31.5%)	2.97 (1.75-5.03)	<0.001	2.41 (1.36-4.28)	**0.003**
Missing	9/36 (25.0%)				
**Receptor group**					
Luminal A	104/526 (19.8%)	Ref.*		Ref.	
Luminal B	65/251 (25.9%)	1.40 (1.03-1.91)	0.033	1.39 (1.01-1.92)	**0.043**
HER2/triple neg	39/140 (27.9%)	1.60 (1.11-2.31)	0.012	1.19 (0.77-1.86)	0.434
Missing	9/33 (27.3%)				

*The proportional hazards assumption was not met, HR should thus be interpreted as average over time.

Supplementary Table 1: Cox proportional hazard regression for all recurrence, 10-year follow-up. *P* values are based on the Wald test; *p* values < 0.05 in bold text.

Although tumor size was significant in the univariable analysis, it did not remain significant in the initial multivariable model including all variables (p = 0.217) and was therefore excluded from the final model. Notably, the prognostic impact of the medium pSMAD2 group remained significant even when tumor size was included in the multivariable model (p = 0.006).

HR Hazard ratio, *CI* confidence interval, *RT* radiotherapy

Supplementary Figure 1

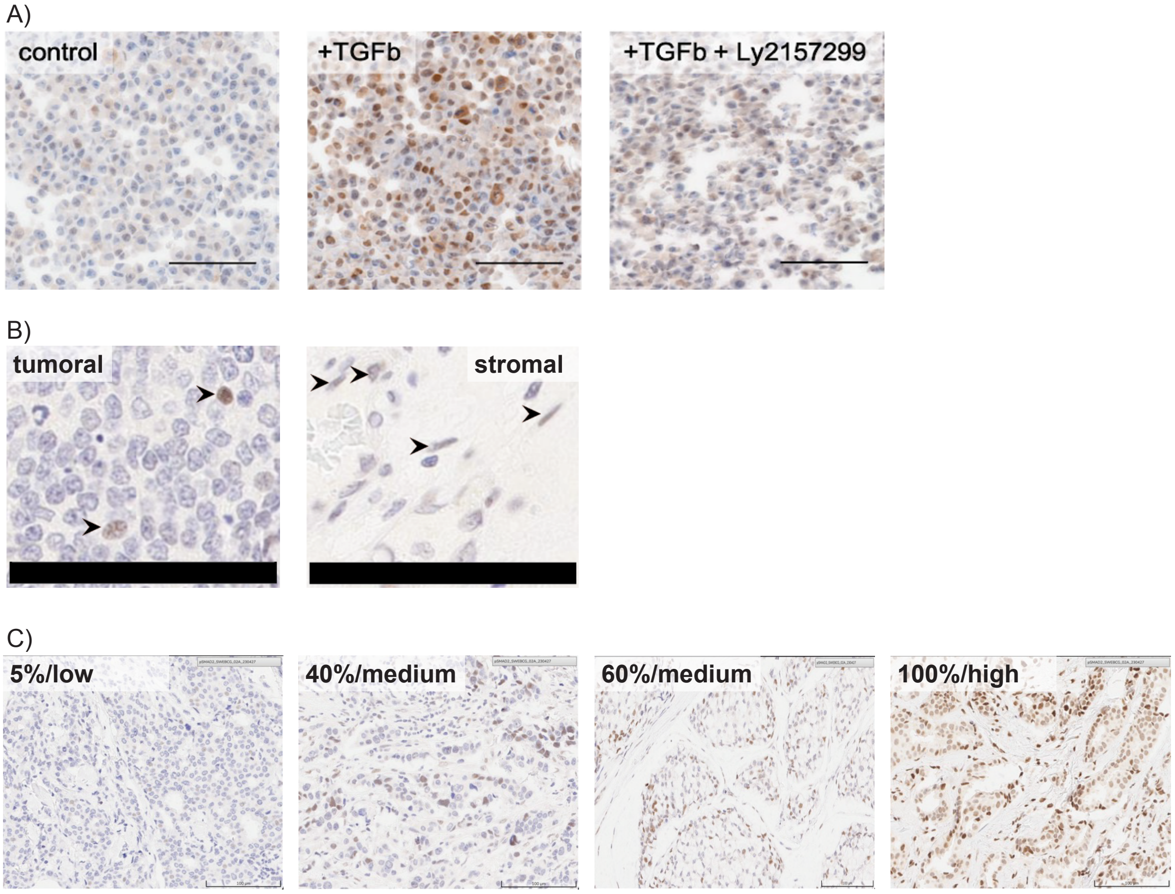

A) pSMAD2 staining controls: Cell cultures (A549) control, +TGFβ (3h) and +TGFβ+Ly2157299 (TGFBR type I inhibitor) were prepared as FFPE blocks following the clinical routine procedures at the pathology unit in order to serve as control samples. Freshly cut sections were stained for pSMAD2 by IHC.
A strong nuclear pSmad2 staining is observed upon TGF-beta stimulation which is reduced in the precense of the inhibitor.
B) Illustration of positive nuclear pSMAD2 staining in tumor (right image) and stroma (left image) cells.
C) Representative image of pSMAD2 IHC staining of samples included in the SweBCG91RT trial.
Scale bars = 100um

Supplementary Figure 2

A)

B)

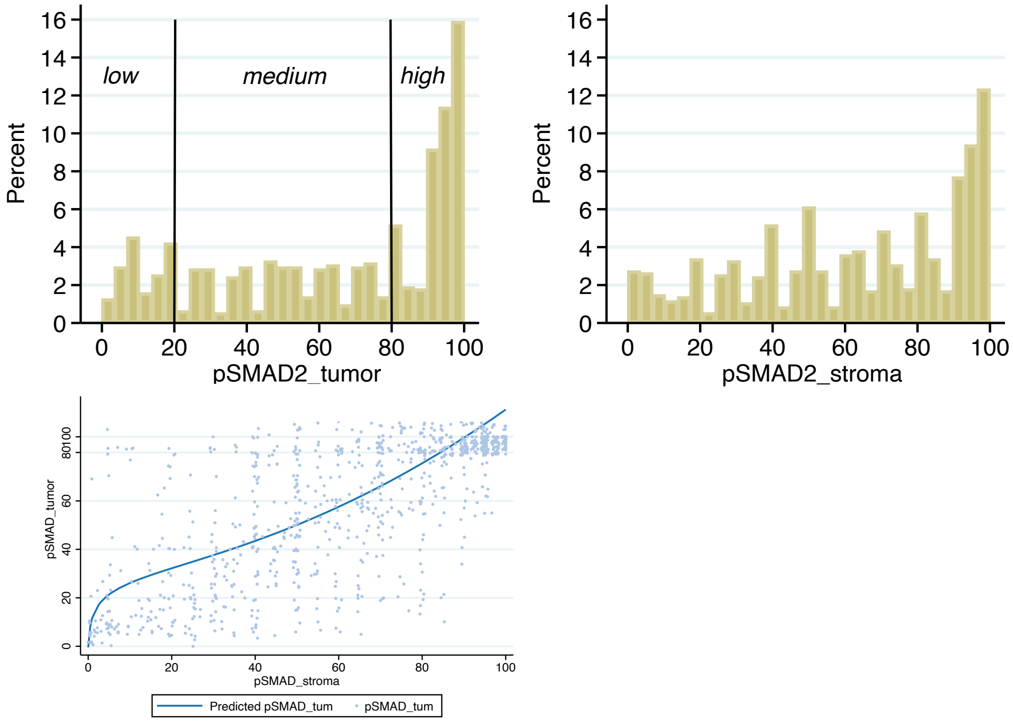

A) Score distribution of the fraction of tumoral (left) and stromal (right) cells positive for nuclear pSMAD2. Based on the distribution pattern, the tumoral pSMAD2 scores were categorized as low (≤20%, n=162), medium (21-79%, n=358) or high (≥80%, n=430) nuclear pSmad2 staining.
B) Correlation between the frequency of pSMAD2 positive fraction in the tumoral and stromal compartment.
